# Low-CO_2_ Optimization Design of Quaternary Binder Containing Calcined Clay, Slag, and Limestone

**DOI:** 10.3390/ma16196385

**Published:** 2023-09-24

**Authors:** Run-Sheng Lin, Yongpang Liao, Yi Han, Seokhoon Oh, Ki-Bong Park, Hyun-Min Yang, Xiao-Yong Wang, Bo Yang, Li-Yi Meng

**Affiliations:** 1 Faculty of Civil Engineering and Mechanics, Kunming University of Science and Technology, Kunming 650500, China; linrunsheng@kust.edu.cn (R.-S.L.); 20222210088@stu.kust.edu.cn (Y.L.); 2 Yunnan Key Laboratory of Disaster Reduction in Civil Engineering, Kunming 650500, China; 3 International Joint Laboratory for Green Construction and Intelligent Maintenance of Yunnan Province, Kunming 650500, China; 4 Department of Integrated Energy and Infra System, Kangwon National University, Chuncheon-si 24341, Republic of Korea; hanyii@kangwon.ac.kr (Y.H.); gimul@kangwon.ac.kr (S.O.); yangbo@kangwon.ac.kr (B.Y.); mengliyi@kangwon.ac.kr (L.-Y.M.); 5 Department of Architectural Engineering, Kangwon National University, Chuncheon-si 24341, Republic of Korea; kbpark@kangwon.ac.kr; 6 Division of Smart Convergence Engineering, Hanyang University ERICA, 1271 Sa-3-dong, Sangnok-gu, Ansan 15588, Republic of Korea

**Keywords:** quaternary composite cement, optimal design, workability, strength, CO_2_ emission

## Abstract

Blended cement is commonly used for producing sustainable concretes. This paper presents an experimental study and an optimization design of a low-CO_2_ quaternary binder containing calcined clay, slag, and limestone using the response surface method. First, a Box–Behnken design with three influencing factors and three levels was used for the combination design of the quaternary composite cement. The lower limit of the mineral admixtures was 0%. The upper limits of slag, calcined clay, and limestone powder were 30%, 20%, and 10%, respectively. The water-to-binder ratio (water/binder) was 0.5. Experimental works to examine workability and strength (at 3 and 28 days) were performed for the composite cement. The CO_2_ emissions were calculated considering binder compositions. A second-order polynomial regression was used to evaluate the experimental results. In addition, a low-CO_2_ optimization design was conducted for the composite cement using a composite desirability function. The objectives of the optimization design were the target 28-day strength (30, 35, 40, and 45 MPa), target workability (160 mm flow), and low CO_2_ emissions. The trends of the properties of optimal combinations were consistent with those in the test results. In summary, the proposed optimization design can be used for designing composite cement considering strength, workability, and ecological aspects.

## 1. Introduction

Clay and limestone are minerals available in many countries worldwide, and granulated blast furnace slag is a byproduct of the steel-making industry. Calcined clay, slag, and limestone are being increasingly used as mineral admixtures in concrete production. Using these mineral admixtures, numerous advantageous properties can be achieved, such as good workability, low hydration heat, and low environmental impact. To rationally use calcined clay, slag, and limestone, both experimental studies and numerical optimization studies of the workability, mechanical, and ecological aspects are necessary [1,2].

Many experimental studies have been conducted on binary or ternary blended concretes containing calcined clay, slag, and limestone. First, regarding binary blended concretes, Kang et al. [3] found that cement hydration can be accelerated by the inclusion of limestone powder. Li et al. [4] reported that the threshold value of chloride ions decreases as the content of limestone increases. Tironi et al. [5] showed that calcination and grinding improve the reaction of raw clay. Lin et al. [6] found that calcined hwangtoh clay has nucleation, dilution, and chemical effects on the hydration reaction of ordinary Portland cement. Kocaba et al. [7] reported that calorimetry calibration and backscattered electron imaging are promising methods to measure the reaction degree of slag. Second, for ternary hybrid concrete, Arora et al. [8] found a synergistic effect between limestone and slag and determined the linear relationship between carbonate consumption and carboaluminate formation. Ramezanianpour and Hooton [9] found that a high content of alumina in slag or metakaolin increases the reaction rate of limestone and its optimum level. Dhandapani and Santhanam [10] showed that cement-based material with a limestone-calcined clay–cement (LC3) binder has much lower permeability than a fly ash hybrid binary concrete. Dhandapani et al. [11] presented results showing that LC3 concretes are suitable for structures in a chloride attack environment. Pillai et al. [12] reported that LC3 concrete has a much lower CO_2_ footprint than plain concrete while having similar strength. In summary, previous experimental studies have mainly focused on the properties of ternary or binary blended concretes; there have been few studies on quaternary blended concretes incorporating calcined clay, slag, and limestone filler [13,14]. In some countries, such as China, Korea, and India, many mineral admixtures are in use, such as slag, calcined clay, and limestone. Concrete producers and researchers are interested in whether slag, calcined clay, and limestone can be used together for producing quaternary blended cement. In addition, what about the performance of such quaternary-blended-cement-based materials? Are there possible synergistic effects among calcined clay, slag, and limestone? 

Several numerical models for blended cement have been proposed to evaluate the properties of blended concretes. First, regarding binary blended concretes, Kolani et al. [15] modeled the kinetic process of hydration of slag–cement binary blends and evaluated the heat of hydration, combined water, and calcium hydroxide. Wang [16] proposed an integrated model for metakaolin–cement hybrid blends and evaluated the degree of reaction of metakaolin and cement, strength, and permeability properties of metakaolin hybrid concretes. Wang [17] evaluated the degree of hydration, strength development, and carbonation durability of limestone-blended concrete considering the nucleation effect and dilution effect of limestone filler. Second, regarding ternary blended concretes, Kunther et al. [18] conducted thermodynamic modeling and evaluated the compositions of reaction products of ternary blends of cement–metakaolin–limestone. Wang and Luan [19] proposed a kinetic hydration and strength model for ternary blends of cement–slag–limestone and determined the optimum combination ratio of slag and limestone for different ages. Carrasco et al. [20] proposed experimental design methods and produced market-oriented cement containing slag and limestone. Yang et al. [21] simulated chloride penetration through LC3 high-performance concretes considering the binder composition and the damage. Avet et al. [22] determined the reacted extent of metakaolin in calcined clay and evaluated the phase assemblage of an LC3 paste by thermodynamic modeling and mass balance. In summary, the current numerical processes are mainly valid for plain, binary, or ternary hybrid concretes, but models that are effective for quaternary blended concretes require further investigation. 

Based on this literature study, we found that compared with fundamental studies on plain, binary, or ternary blended concretes, studies on quaternary concretes are limited. To fill this gap, this paper presents experimental studies and numerical modeling of quaternary cement containing slag, calcined clay, and limestone. Tests of the workability and strength were performed, and the CO_2_ emissions were calculated. Furthermore, based on the test results, a numerical model was used to determine the optimum combinations with target flow and strength and low CO_2_ emissions.

Compared with previous research, the scientific innovations of this article mainly include the following aspects: 1. The research object is four mixed cement-based materials. Previous studies were mostly on two and three mixed cement-based materials; 2. The response surface design method proposed in this paper systematically considers fluidity, strength, and CO_2_ emissions, while CO_2_ emissions were not considered in previous concrete mix design methods; 3. The design method proposed in this article has certain portability and is suitable for designing low-carbon concretes in different countries and regions.

## 2. Materials and Methods

### 2.1. Materials

**Figure 1:**
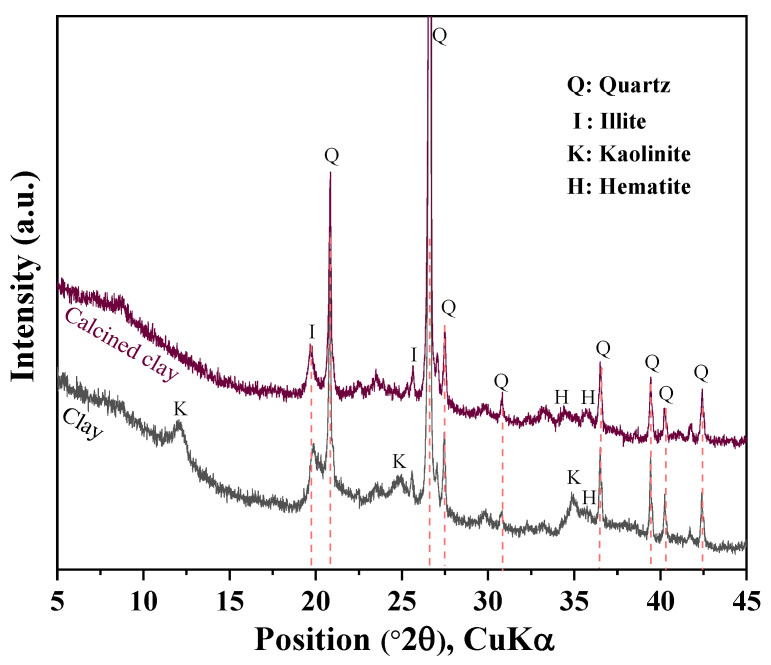
XRD analysis of calcined clay and raw clay.

Ordinary Portland cement of ASTM type I (ASTM C150) was obtained from Sung Shin Cement [23], Republic of Korea. Hwangtoh is a clay mineral widely distributed in Korea. It contains high levels of calcium potassium chloride and calcium. Clay was ground before calcination, and calcined clay was produced by calcining hwangtoh clay at 800 °C for 60 min [6]. Figure 1 shows the XRD analysis results of clay before and after calcination. We can see that the calcination eliminated the kaolinite peak (2θ = 12.37°) [6]. Limestone powder and granulated blast furnace slag were obtained from Daejung Chemical Co., Ltd. (Sinan, Republic of Korea) and Asia Cement Co., Ltd. (Seoul, Republic of Korea), respectively. Table 1 presents the chemical compositions of the cement, limestone, slag, and calcined clay. The standard deviation for the XRF analysis results in Table 1 is 0.01%. Calcined clay and slag have higher aluminum contents than cement, which can support a synergistic effect between the limestone and slag or calcined clay. Calcined clay presents a higher silicon content than cement, which can contribute to the production of secondary calcium silicate hydrate (CSH). The average particle sizes of the cement, slag, calcined clay, and limestone were 18.3 µm, 12.8 µm, 12.3 µm, and 7.4 µm, respectively.

### 2.2. Experimental Method

Mixture preparation: The cement paste was mixed using a mechanical mixer and immediately put into a steel mold. At the age of 1 day, the steel mold was removed, and after removal, the paste specimens were placed in a curing chamber for sealed curing until they reached test age. The temperature for sealed curing was 20 degrees Celsius.

Test: The flow properties of the slurries were measured based on the mini-slump test method. The dimensions of the mini-slump cone were a height of 50 mm, a top diameter of 70 mm, and a bottom diameter of 100 mm. In addition, at curing ages of 3 days and 28 days, the compressive strength of the paste samples was tested according to ASTM C349 [24]. The sample size for compressive strength testing was 50 × 50 × 50 mm. Samples for compressive strength testing were cured via sealed curing. For each mixture, three specimens were prepared. The average of the measured values for the three specimens was taken as the strength value for each group of specimens. However, when the difference between the maximum or minimum value among the three measured values and the middle value exceeded 15% of the middle value, the maximum and minimum values were eliminated, and the middle value was taken as the compressive strength of the group of specimens.

### 2.3. Experimental Design

**Figure 2:**
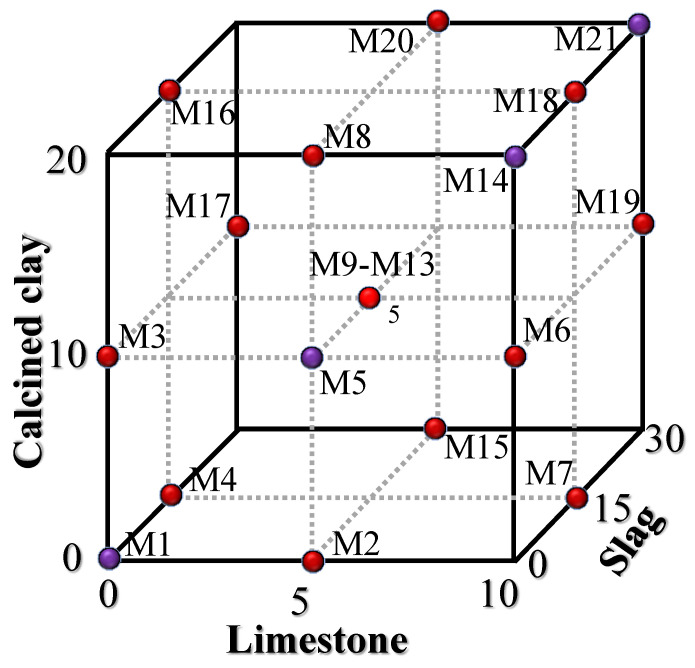
Combination design of 21 specimens (17 specimens from BBD (red points) and 4 additional specimens (purple points)).

A Box–Behnken design (BBD) was employed to design the quaternary hybrid cement combinations [25,26]. As can be seen from Table 2, the BBD consisted of three independent factors (limestone, calcined clay, and slag). For each factor, there were three values corresponding to codes +1, 0, and −1. The maximum replacement ratios (high-coded +1) of limestone, calcined clay, and slag were 10%, 20%, and 30%, respectively. The maximum replacement ratio by the mineral admixture was 60% (10% + 20% + 30% = 60%). Cases of control, binary blends, ternary blends, and quaternary blends were considered. The minimum ratio (low-coded −1) for limestone, calcined clay, and slag was 0%. The water-to-binder ratio of the specimens was 0.5. The combinations of components of the binders are shown in Figure 2 and listed in Table 3. A total of 21 specimens were prepared, consisting of 17 BBD specimens and 4 additional specimens. The additional specimens were added to consider the lower limit combination (M1), the upper limit combination (M21), and the synergetic effect between limestone and calcined clay (M5 and M14).

CO_2_ emissions are an important indicator of sustainable concrete. Herein, quaternary cement was designed with CO_2_ emissions as the optimization target. According to the sample mixtures and the specific CO_2_ emissions of each binder (listed in Table 4) [27], the CO_2_ emissions per unit volume of each sample can be determined.

The CO_2_ emissions of 1 m^3^ of composite paste can be determined as follows. First, we determine the individual masses of the paste components:

(1) $$\sum_{i=1}^{5}\frac{m_{i}}{\rho_{i}}=1$$

where $m_{i}$ and $\rho_{i}$ are the mass and density (shown in Table 1), respectively, of component *i* of the composite binder. Because the composite binder consists of five constituents, i.e., cement, calcined clay, slag, limestone powder, and water, the value of subscript *i* ranges from 1 to 5. Second, from the individual masses and individual CO_2_ emissions of the components of paste (shown in Table 4), we can calculate the CO_2_ emissions of 1 m^3^ of paste.

(2) $$CO_{2}=\sum_{i=1}^{5}m_{i}\times CO_{2}{}_{i}$$

Here, $CO_{2}{}_{i}$ is the individual *CO*_2_ emissions (shown in Table 4) of component *i* of the paste, and $CO_{2}$ is the total *CO*_2_ emissions of 1 m^3^ of paste. 

A second-order polynomial model was employed to perform a regression of the results (flow, strength, and *CO*_2_ emissions) and examine the effect of the type and content of the mineral admixture [26]. The general equation of the second-order polynomial regression model is as follows:

(3) $$y=\beta_{0}+\sum_{i=1}^{3}\beta_{i}x_{i}+\sum_{i=1}^{3}\beta_{ii}x_{i}^{2}+\sum_{i=1}^{2}\sum_{j>i}^{3}\beta_{ij}x_{i}^{}x_{j}^{}$$

where *y* is an experimental result (such as flow, strength, or *CO*_2_ emissions), $x_{i}$ is an independent factor (three independent factors were employed, i.e., the contents of limestone, calcined clay, and slag), $\beta_{0}$ is the intercept coefficient, $\beta_{i}$ is the linear term coefficient, $\beta_{ii}$ is the quadratic term coefficient, and $\beta_{ij}$ is the coefficient of the interaction term. For different experimental tests, the coefficients ($\beta_{0}$, $\beta_{i}$, $\beta_{ii}$, and $\beta_{ij}$) may differ.

## 3. Experimental Results

The experimental results (strength, flow) and CO_2_ emission calculation results are summarized in Table 5. Table 6 shows the regression coefficients of all the results. As shown in Table 6, the parameters of the equations consist of an intercept, linear term, quadratic terms, and interaction terms. Based on the results for flow, strength, and CO_2_ emissions, the coefficients of the equations were calibrated. The proposed equations can be validated by examining the *p*-value, lack of fit, and R^2^ of the regression. The coefficients of determination between the experimental and predicted values were higher than 0.95, which means that the proposed equations for regression of the results are reasonable. The *p* values of the regression equations were less than 0.0001, which suggests that these regression models are significant. In addition, the lack of fit for each result was not significant relative to the pure error.

### 3.1. Flow of Paste

**Figure 3:**
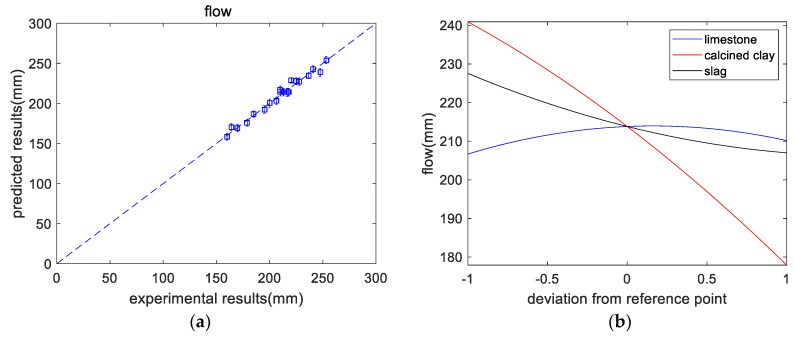
Results of flow analysis. (**a**) Experimental versus predicted results. (**b**) Perturbations of flow.

As shown in Table 5, the overall trend of flow is that after adding SCM, the flow of the paste was reduced. The flow of M21 was the lowest, at 160 mm, mainly because M21 contains a high content of clay and slag. These two substances have high aluminum contents, which can reduce the flow. From the experimental results (shown in Figure 3a), the flow of a paste was regressed as follows:

(4) $$\begin{array}{l} flow=213.82+1.75*x_{1}-31.50*x_{2}-10.28*x_{3}-5.39*{\left(x_{1}\right)}^{2}-4.39*{\left(x_{2}\right)}^{2}+3.47*{\left(x_{3}\right)}^{2}\\ -5.18*x_{1}*x_{2}-3.13*x_{1}*x_{3}-0.63*x_{2}*x_{3} \end{array}$$

where $x_{1}$, $x_{2}$, and $x_{3}$ denote the contents of limestone, calcined clay, and slag, respectively. The predictions of flow agreed with the experimental results. Figure 3b shows the impact of each component on flow. Limestone has a negligible effect on flow. With increasing slag content, the flow rate decreases slightly. This is because of the higher surface area of slag than cement. However, as the content of calcined clay increases, the flow decreases significantly. This is because of the higher aluminum content in calcined clay than in cement. A higher aluminum content makes the mixture prone to forming ettringite, which can lower the flow [28]. In addition, the high surface area and specific structure of calcined clays also contribute to a lower slump. Likewise, Lin et al. [6] found that the addition of calcined hwangtoh clay can significantly reduce the slump of concrete.

### 3.2. Compressive Strength

**Figure 4:**
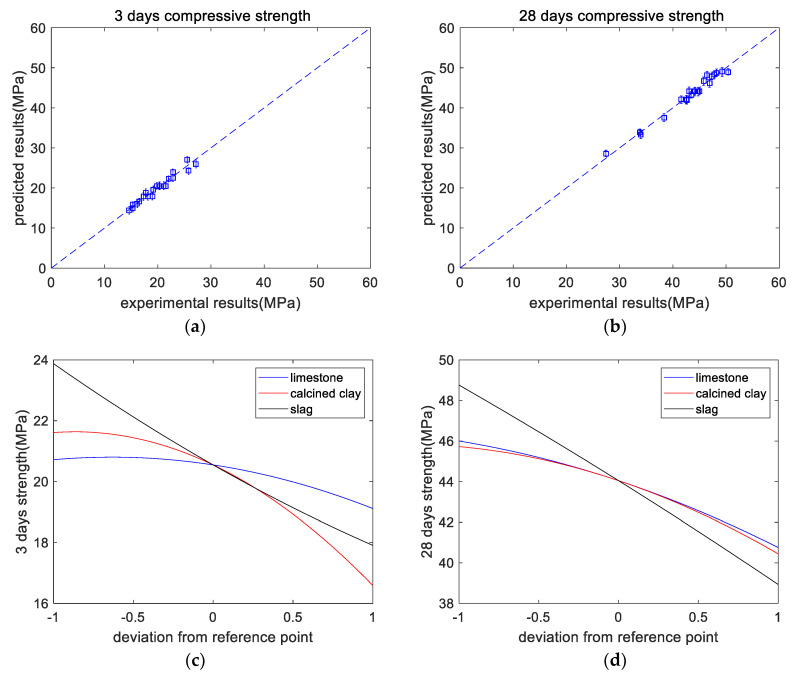

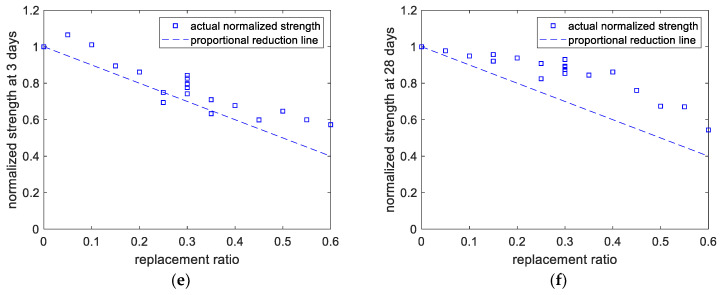
Results of compressive strength analysis. (**a**) Experimental versus predicted results of 3-day strength. (**b**) Experimental versus predicted results of 28-day strength. (**c**) Perturbations of variables of 3-day strength. (**d**) Perturbations of variables of 28-day strength. (**e**) Normalized 3-day strength. (**f**) Normalized 28-day strength.

Table 5 shows the overall trend of the 3-day strength (shown in Figure 4a): after adding SCM, the strength of the paste was reduced. This is mainly because of the dilution effect of SCM. M21 had the lowest 3-day strength, which is due to the higher SCM content in M21. In addition, M2 showed slightly higher strength than the control specimen M1, which is due to the limestone powder’s nucleation effect, which accelerates the early-age hydration rate of Portland cement and improves its strength. From the experimental results, the 3-day strength of a paste was regressed as follows:

(5) $$\begin{array}{l} 3\mathrm{days}\mathrm{strength}=20.55-0.80*x_{1}-2.51*x_{2}-2.98*x_{3}-0.63*{\left(x_{1}\right)}^{2}-1.45*{\left(x_{2}\right)}^{2}+0.34*{\left(x_{3}\right)}^{2}\\ +0.65*x_{1}*x_{2}+0.17*x_{1}*x_{3}+1.08*x_{2}*x_{3} \end{array}$$

As shown in Table 5, the overall trend of the 28-day strength was that after adding SCM, the strength of the paste was reduced. This is mainly because of the dilution effect of SCM. M21 had the lowest 28-day strength, which is due to the higher SCM content in M21. In addition, the strength of M2 was slightly lower than that of the standard specimen M1, which differs from the trend for the 3-day strength. This is because the dilution effect of limestone is the dominant factor in the later stage (28 days), while the nucleation effect is the dominant factor in the early stage (3 days). The 28-day strength of a paste (shown in Figure 4b) was determined as follows:

(6) $$\begin{array}{l} 28\mathrm{days}\mathrm{strength}=44.05-2.62*x_{1}-2.64*x_{2}-4.91*x_{3}-0.66*{\left(x_{1}\right)}^{2}-0.96*{\left(x_{2}\right)}^{2}-0.20*{\left(x_{3}\right)}^{2}\\ +0.40*x_{1}*x_{2}-2.37*x_{1}*x_{3}-1.45*x_{2}*x_{3} \end{array}$$

As shown in Figure 4c, at the curing age of 3 days, when the limestone content ranges from 0% to 5% (code changes from −1 to 0), the decrease in the strength is not significant. This is because limestone has a nucleation effect on cement hydration, can improve the hydration extent of cement, and can enhance the early-age strength of concrete. However, when the content of limestone powder further increases from 5% to 10% (code 0 to code +1), owing to the limestone’s dilution effect, the reduction in the strength becomes remarkable. In addition, as displayed in Figure 4d, at the age of 28 days, the strength decreases with increasing mineral admixture. This is due to the dilution effect and the lower reactivity of the mineral admixture compared with that of the cement. It should be noticed that in Figure 4d, the code +1 for limestone and calcined clay represents 10% and 20%, respectively. In other words, for limestone and calcined clay, when the *x*-axis represents the replacement ratio, the regression curves will be different. Figure 4e,f show the normalized strengths at the ages of 3 and 28 days, respectively. The paste’s normalized strength was calculated as the compressive strength ratio between hybrid paste and plain paste. The proportional reduction line corresponds to the dilution effect. At a curing age of 3 days, the normalized strength values are close to the dilution effect line, whereas, at the age of 28 days, they are much higher than the dilution effect line. This indicates the increasing reaction extent of the mineral admixtures from curing ages of 3 days to 28 days.

### 3.3. CO_2_ Emissions

**Figure 5:**
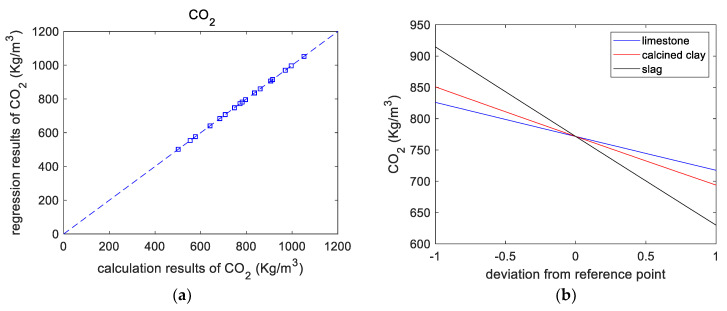

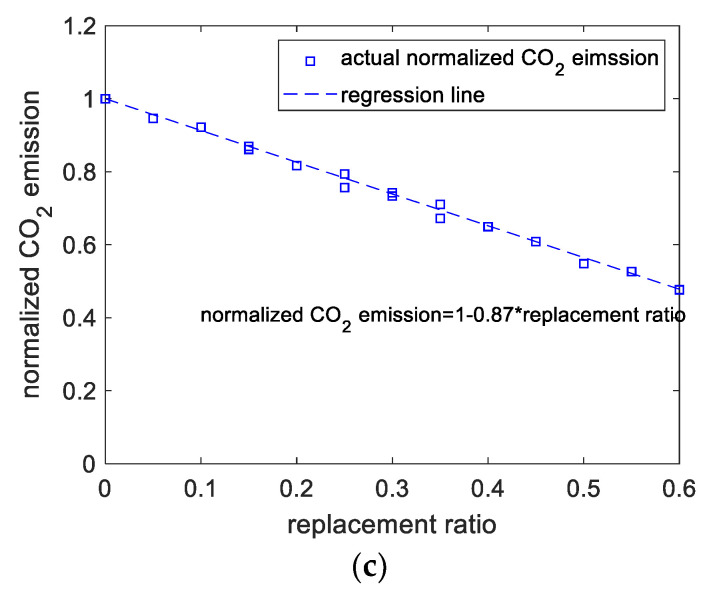
Results of CO_2_ emissions analysis. (**a**) Calculated versus regressed results of CO_2_ emissions. (**b**) Perturbations of variables of CO_2_ emissions. (**c**) CO_2_ emissions versus replacement ratio.

Table 5 shows the overall trend of CO_2_ emissions: after adding SCM, CO_2_ emissions were reduced. This is mainly because of the dilution effect of SCM reducing the amount of cement used. M21 has the lowest CO_2_ emissions due to its higher SCM substitution. From the CO_2_ emission calculation results (shown in Figure 5a), the CO_2_ emissions of a paste were regressed based on a second-order polynomial as follows:

(7) $$\begin{array}{l} {\mathrm{CO}}_{2}=771.62-54.26*x_{1}-78.48*x_{2}-142.45*x_{3}+0.22*{\left(x_{1}\right)}^{2}+0.78*{\left(x_{2}\right)}^{2}+0.70*{\left(x_{3}\right)}^{2}\\ +0.86*x_{1}*x_{2}+0.84*x_{1}*x_{3}+1.80*x_{2}*x_{3} \end{array}$$

Figure 5b presents the effect of the components on CO_2_ emissions. As the individual replacement ratios of the binder components increase, the CO_2_ emissions linearly decrease. It should be noticed in Figure 5b that the code +1 for limestone and calcined clay represents 10% and 20%, respectively. In other words, for limestone and calcined clay, when the *x*-axis represents the replacement ratio, the regression curves will be different. Figure 5c shows the normalized CO_2_ emissions of the blended slurry. The normalized CO_2_ emissions are the ratios of the CO_2_ emissions of blended paste to those of control paste. Figure 5c shows that, for various mixtures, normalized CO_2_ emissions decrease linearly with increasing total substitution rate. Therefore, adding SCMs is an effective way to make low-CO_2_ concrete.

## 4. Optimal Design Results and Discussion

### 4.1. Multi-Objective Optimal Design

The optimized design had multiple goals: target strength and flow values and low CO_2_ emissions. Desirability functions were used for the optimization. Equation (8) shows that the expected value is dependent on the response value [29], with the value of the desirability function ranging from zero to one.

(8) $$0\leq d_{i}(Y_{i})\leq 1$$

Here, $Y_{i}$ and $d_{i}$ are the response value and the desirability function, respectively. Unity and zero correspond to fully achieved and not achieved optimization objectives, respectively.

For multi-objective optimization, based on the individual desirability functions, the composite desirability function can be determined using the following equation [25,29]:

(9) $$D={\left(d_{1}^{r_{1}}*d_{2}^{r_{2}}*....d_{n}^{r_{n}}\right)}^{1/\sum_{i=1}^{i=n}r_{i}}={\left(\prod_{i=1}^{n}d_{i}^{r_{i}}\right)}^{1/\sum_{i=1}^{i=n}r_{i}}$$

where D, *n*, and *r* are the composite desirability function, the total number of optimization objections, and the relative importance of each response, respectively.

Ecological aspects, such as low CO_2_ emissions, are an important aim of optimal design. When the minimum value is set as the aim of optimization, the desirability can be determined using the following equation [25]:

(10) $$d_{i}(Y_{i})=\left\{ \begin{array}{l} 1\begin{array}{cc} & Y_{i}\leq L_{i} \end{array}\\ {\left(\frac{U_{i}-Y_{i}}{U_{i}-L_{i}}\right)}^{s}\begin{array}{cc} & L_{i}\leq Y_{i}\leq U_{i} \end{array}\\ 0\begin{array}{cc} & Y_{i}\geq U_{i} \end{array} \end{array}\right.$$

where L and U denote the lower limit and upper limit of the response value, respectively, and *S* denotes the weight factor. When the value of *S* is equal to 1, Equation (10) is a linear equation. We choose *S* > 1 when closeness to the target value is more important; we choose 0 < *S* < 1 when it is less important. Equation (10) shows that desirability equals 1 when the value of the response is below the lower bound. Desirability equals 0 when the value of the response is above the upper limit.

As shown in Table 5, through the combination of the four binder components, different responses, such as flow and 28-day strength, can be achieved. This can be achieved by optimizing the design. When a range is set as the aim of optimization, the desirability can be determined using the following equation:

(11) $$d_{i}(Y_{i})=\left\{ \begin{array}{l} 0\begin{array}{cc} & Y_{i}<L_{i} \end{array}\\ 1\begin{array}{cc} & L_{i}\leq Y_{i}\leq U_{i} \end{array}\\ 0\begin{array}{cc} & Y_{i}>U_{i} \end{array} \end{array}\right.$$

This equation shows that the desirability function is equal to 1 when the response is between the lower bound and upper bound, and it is 0 when the response is outside these bounds.

The optimization goals are listed in Table 7. The independent variable—the content of limestone, calcined clay, and slag—should be within the lower and upper limits. The variables of the response are as follows: (1) The 28-day strength should not be less than the target strength. In this study, the 28-day target strength values were set to 30, 35, 40, and 45 MPa. (2) The flow should be not less than the target flow (160 mm). (3) The CO_2_ emissions should be as low as possible for purposes of ecofriendliness. In this study, Design Expert software (version 12) was used for the optimization design [29]. The relative importance r and weighting factor S of all responses were set to 1 [26].

In summary, we consider four optimal design cases with different design strengths. Each design case considers basic requirements (strength and workability) and an ecological factor (CO_2_ emissions). Multi-objective optimization was implemented based on a composite desirability function, which was calculated using the responses and individual desirability.

### 4.2. Results of Optimal Design

The results of the design case combinations are identified and summarized in Table 8. Mix-30, Mix-35, Mix-40, and Mix-45 have 28-day strengths of 30, 35, 40, and 45 MPa, respectively. Table 9 shows that the values of strength and flow for Mix-30, Mix-35, Mix-40, and Mix-45 meet the design requirements.

**Figure 6:**
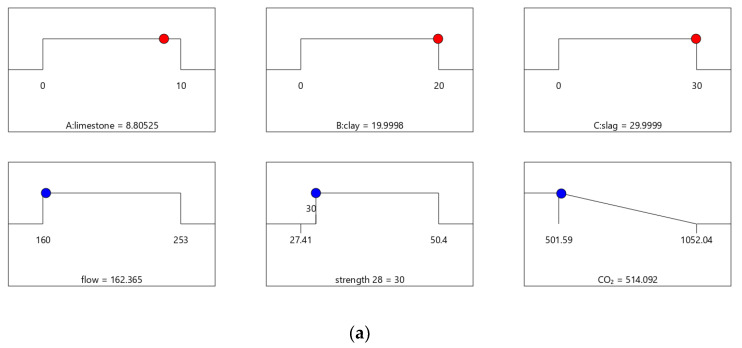

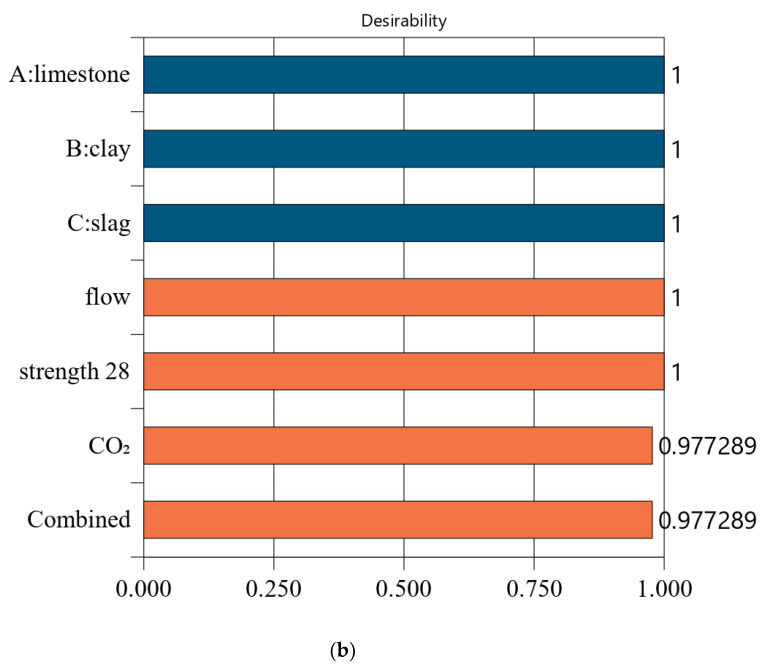
Properties of Mix-30. (**a**) Independent variables and responses of Mix-30. (**b**) Desirability of Mix-30.

**Figure 7:**
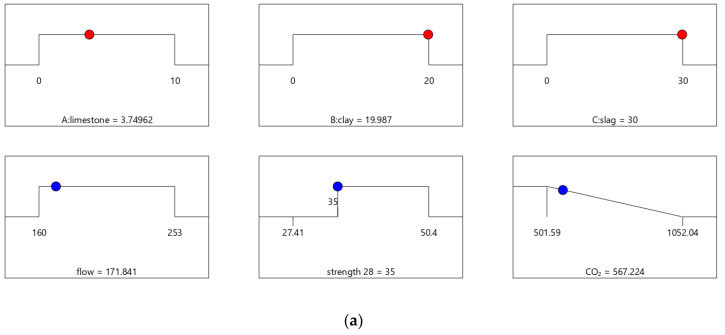

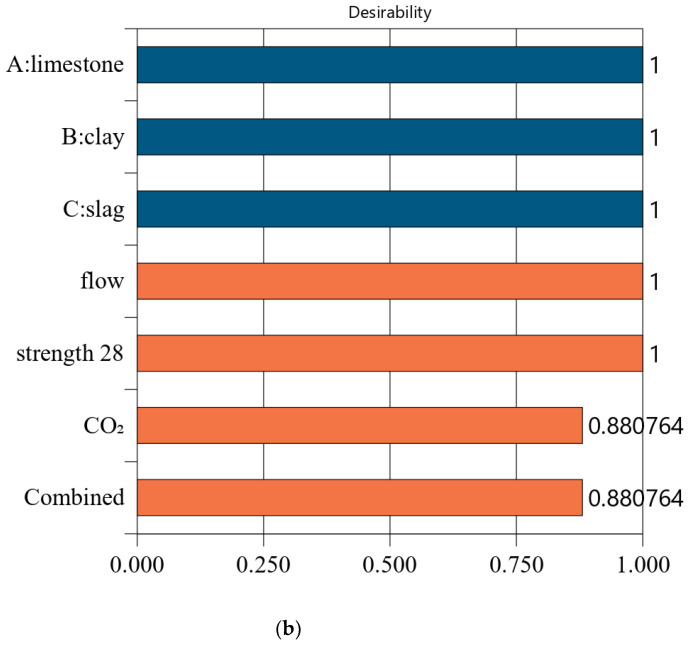
Properties of Mix-35. (**a**) Independent variables and responses of Mix-35. (**b**) Desirability of Mix-35.

**Figure 8:**
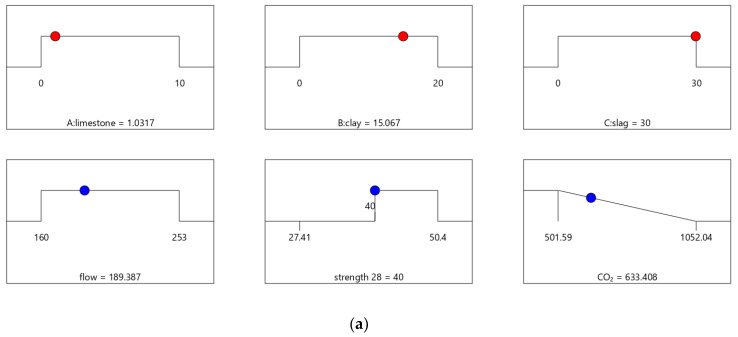

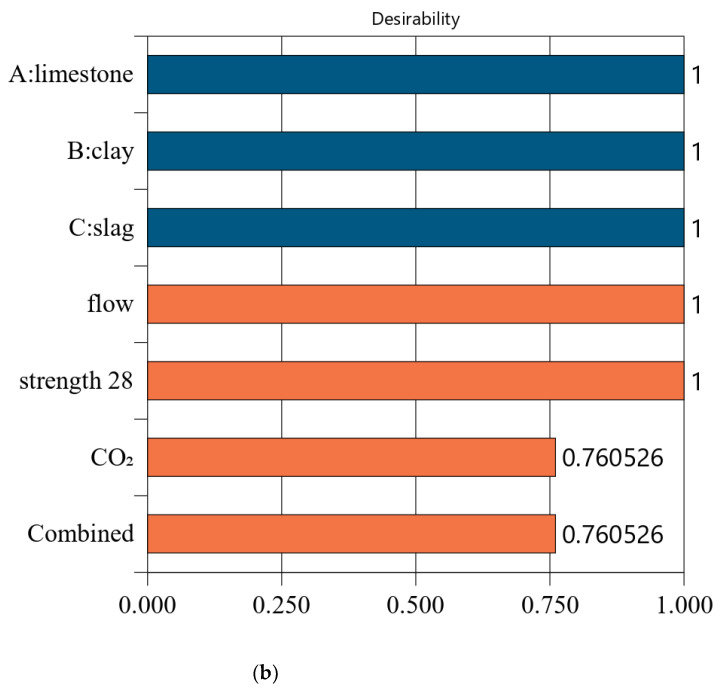
Properties of Mix-40. (**a**) Independent variables and responses of Mix-40. (**b**) Desirability of Mix-40.

**Figure 9:**
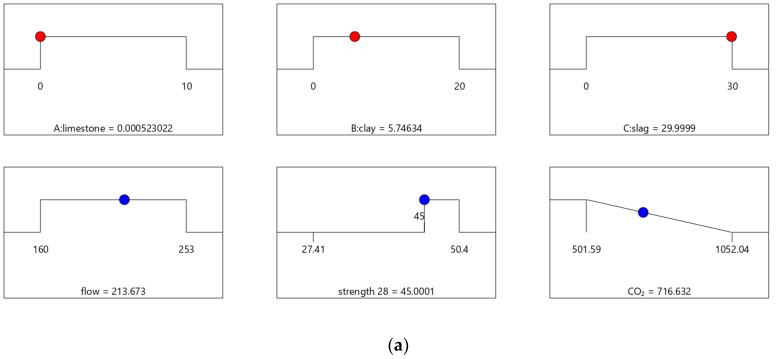

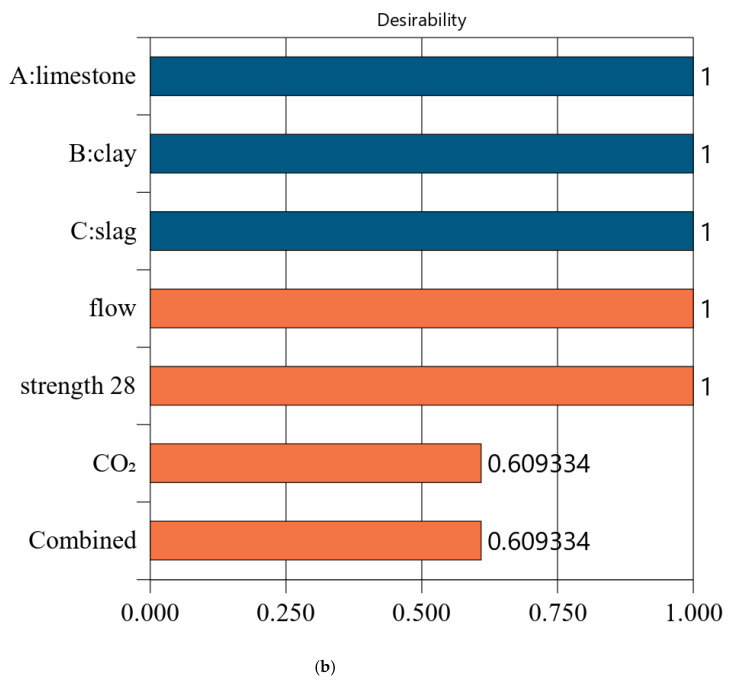
Properties of Mix-45. (**a**) Independent variables and responses of Mix-45. (**b**) Desirability of Mix-45.

Figure 6, Figure 7, Figure 8 and Figure 9 show the independent variables (limestone, calcined clay, and slag) and responses (strength, flow, and CO_2_ emissions) of Mix-30 to Mix-45, respectively. In Figure 6a, Figure 7a, Figure 8a, and Figure 9a, the first row represents limestone, clay, and slag contents, and the second row represents the results of multi-objective optimization (such as flow, strength, and CO_2_ emissions). The optimized flow and strength meet the constraints of the target range, and the optimized CO_2_ emissions are the minimum value. From the minimum value of 501.59 to the maximum value of 1052.04, the CO_2_ optimization decreases with the falling line. This means that the closer the CO_2_ emissions are to the minimum value of 501.59, the higher the satisfaction degree of the optimization; on the contrary, a lower optimization satisfaction degree occurs when the CO_2_ emissions are closer to the maximum value of 1052.04. Figure 6a shows that the limestone, calcined clay, and slag contents of Mix-30 are 8.81%, 20%, and 30%, respectively. Solid red circles in the first row of Figure 6a show the component values of limestone, clay, and slag. The content of the mineral admixtures is close to the upper limit. Solid blue circles in the second row of Figure 6a show the response values for strength, flow, and CO_2_ emissions. The strength and flow rate both meet the design requirements. The CO_2_ emissions are close to a minimum. Figure 6b shows the desirability of the flow, 28-day strength, and CO_2_ emissions, as well as the composite desirability. The desirability is 1 for both the 28-day strength and flow. The composite desirability value for Mix-30 is 0.977.

Figure 7 shows the desirability of Mix-35. The limestone, calcined clay, and slag contents of Mix-35 are 3.75%, 19.98%, and 30.00%, respectively (see the first row in Figure 7a). Since the replacement rate of mineral admixture in Mix-35 is smaller than that in Mix-30, Mix-35 has higher CO_2_ emissions than Mix-30. The desirability is 1 for both the strength and flow, but the CO_2_ emissions are less desirable than those for Mix-30. This is because as the strength of the concrete increases, its carbon dioxide emissions also increase, resulting in a decrease in the desirability function corresponding to CO_2_ emissions. In addition, Mix-35 has a lower composite desirability than Mix-30 (as shown in Figure 6b and Figure 7b).

The desirability values of Mix-40 and Mix-45 are shown in Figure 8 and Figure 9, respectively. Compared to Mix-30, Mix-35, and Mix-40, the total substitution rate of mineral admixture in Mix-45 is much lower. Therefore, Mix-45 has lower CO_2_ emissions than the other cases (as shown in Figure 8b). Consequently, the composite desirability of CO_2_ emissions for Mix-45 is also lower than those for the other cases. 

### 4.3. Optimized Design of CO_2_ Emissions Based on 1 kg Binder

In the optimal design method shown in Section 4.2, the CO_2_ emission is based on each cubic meter of cement paste. In actual engineering, CO_2_ is often calculated based on each kilogram of binder. Based on the mix ratio and the CO_2_ emissions of binder components in Table 4, the CO_2_ emissions per kilogram of binder can be calculated (shown in Table 10). Moreover, the CO_2_ emission of 1 kg binder can be determined using a linear equation as follows:

(12) $${\mathrm{CO}}_{2}\;\mathrm{of}\;\mathrm{binder}=0.86-0.00852*x_{1}-0.0059*x_{2}-0.0077*x_{3}$$

**Figure 10:**
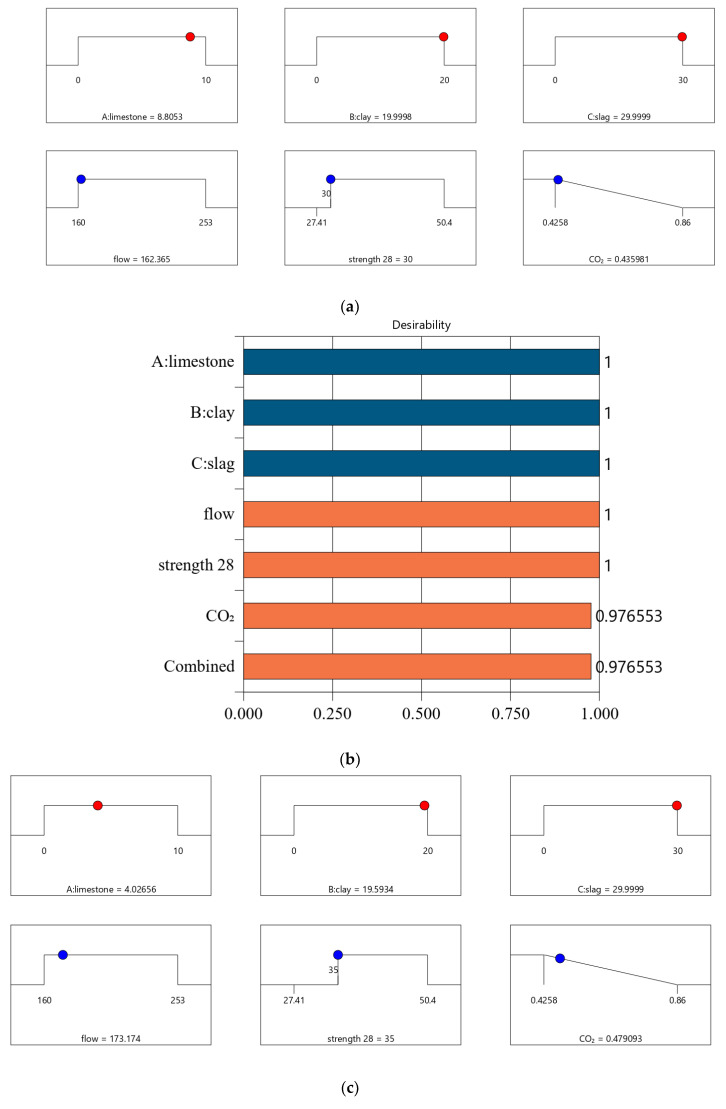

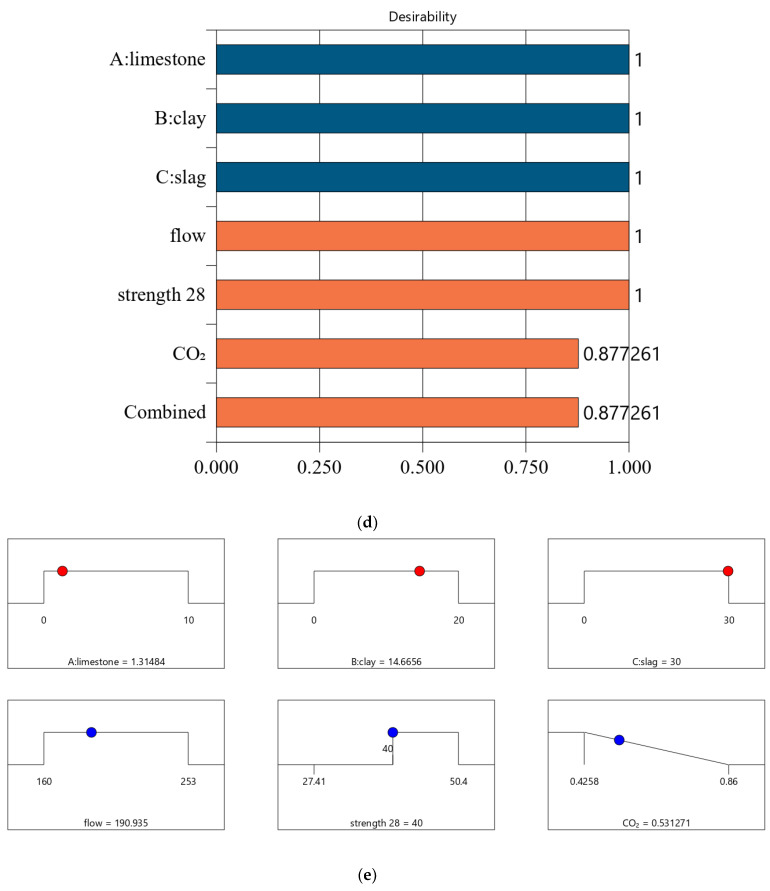

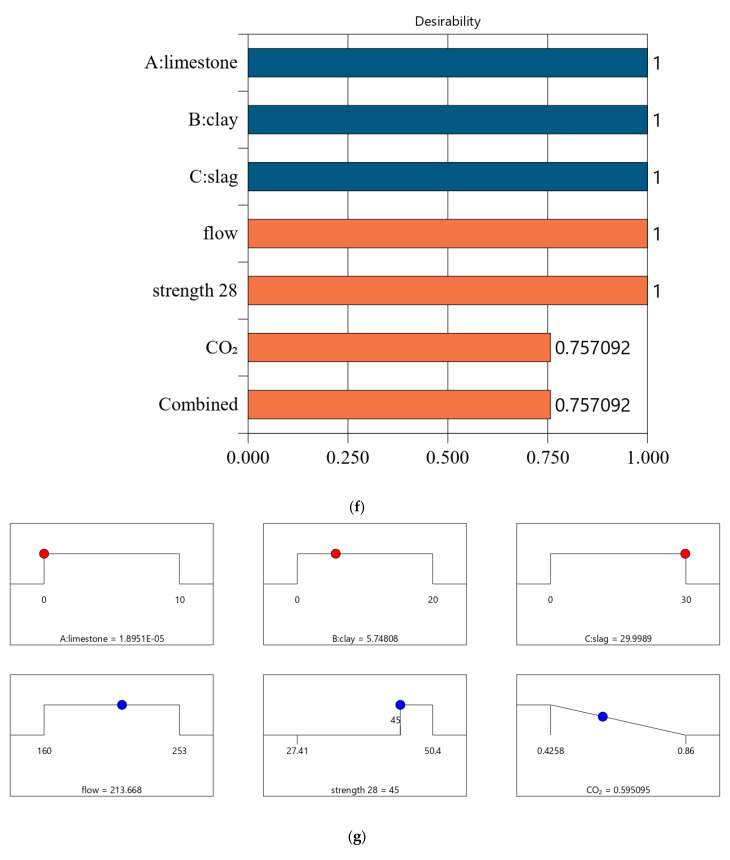

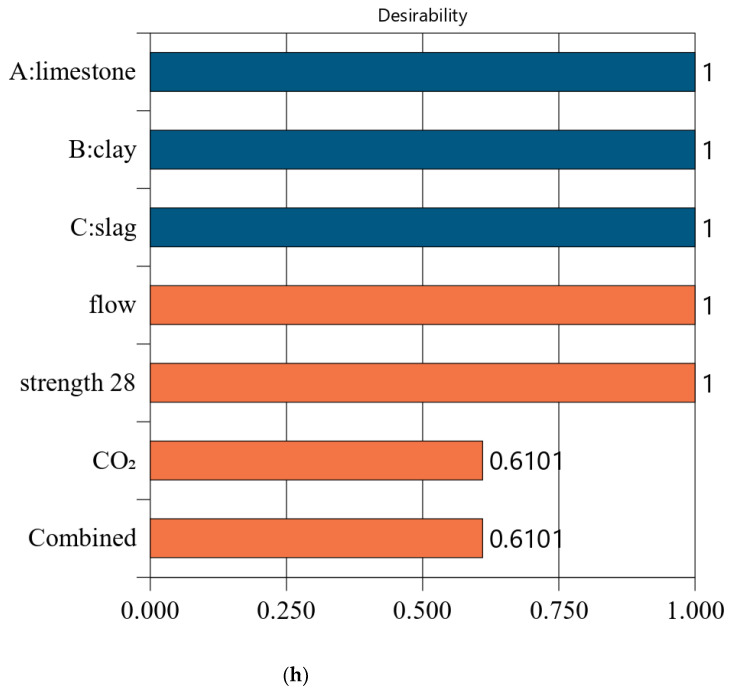
Results of optimal design for CO_2_ emission of 1 kg binder. (**a**) Independent variables and responses of Mix-30 (CO_2_ emission of 1 kg binder). (**b**) Desirability of Mix-30 (CO_2_ emission of 1 kg binder). (**c**) Independent variables and responses of Mix-35 (CO_2_ emission of 1 kg binder). (**d**) Desirability of Mix-35 (CO_2_ emission of 1 kg binder). (**e**) Independent variables and responses of Mix-40 (CO_2_ emission of 1 kg binder). (**f**) Desirability of Mix-40(CO_2_ emission of 1 kg binder). (**g**) Independent variables and responses of Mix-45 (CO_2_ emission of 1 kg binder). (**h**) Desirability of Mix-45 (CO_2_ emission of 1 kg binder).

Following a similar method in Section 4.2, we conducted optimized designs for different strength levels, and the calculation results are shown in Figure 10. The combination of the mix ratio and performance of optimized designs are shown in Table 11 and Table 12, respectively. According to these calculation results, we can find: First, as shown in Table 11, as the design strength increases, the cement content increases. As shown in Table 12, as the design strength increases, the CO_2_ emissions also increase. These trends are consistent with that in Section 4.2. Second, for Mix-30, Mix-35, Mix-40, and Mix-45, the overall satisfaction degree is 0.977, 0.877, 0.757, and 0.610, respectively. Comparing the overall satisfaction degree results in Section 4.2 and the satisfaction degree in this section, we can find that the satisfaction degree values are similar for CO_2_ emissions per cubic meter of paste and CO_2_ emissions per kilogram of binder. Overall, the design method proposed in this article has certain versatility and can be suitable for different optimization objectives.

### 4.4. Discussion of the Optimal Design

In traditional design processes, concrete mix proportional design considers only strength and workability [17,30,31]. However, with the development of concrete techniques, customers demand not only strength and workability but also better ecological performance, such as with regard to CO_2_ emissions [32,33]. The model presented in this study considers more objectives than the conventional mix design approaches, which is important for performance-based design. 

In traditional response surface optimization design, all optimization goals should be measured based on experimental results. However, there have been some new developments, and some researchers have adopted the response surface method to optimize concrete materials. In prior studies, the mechanical properties of the materials were measured experimentally, and the cost and CO_2_ emissions of concrete materials were calculated based on the mix ratio [33,34]. Iman and Camoes [34] measured the strength and workability of ultra-high-performance concrete (UHPC), calculated the cost and CO_2_ emissions of UHPC using concrete components, and produced cost-efficient or CO_2_-efficient optimal designs using the response surface method. Similarly, Mohammad and Ozgur [33] measured the strength of UHPC, calculated the cost of UHPC, and determined optimal designs with a low cost. The rationality of these studies was verified by experiments. Inspired by these studies, we adopted a similar method. The strength and flow were measured experimentally, and the CO_2_ emissions were calculated using a formula. 

In addition, in the experimental design of this paper, the water used was distilled water. More recently, wastewater has also begun to be used in concrete materials. It is necessary to pay attention to the compositional differences between wastewater and distilled water and the resulting impact on the physical and chemical properties of concrete. The response surface design method proposed in this paper is suitable not only for distilled water concrete but also for wastewater concrete.

## 5. Conclusions

This study presents experimental works and multi-objective optimization design regarding a quaternary hybrid cement containing calcined clay, slag, and limestone. A total of 21 mixtures were prepared, of which 17 were BBD samples and 4 were additional samples. The flow, compressive strength (at 3 and 28 days), and CO_2_ emissions of the various mixtures were determined. The following conclusions were obtained based on the test results:

(1) Flow, strength, and CO_2_ emissions decrease with increasing mineral admixture content. The flow decreases significantly with increasing calcined clay content. At 3 days of age, the normalized strength value is close to the dilution effect line, but at 28 days of age, the normalized strength value is much higher than the dilution effect line. Normalized CO_2_ emissions decrease linearly as the substitution rate increases.
(2) Four design cases (Mix-30, Mix-35, Mix-40, and Mix-45) with different 28-day design strengths (30, 35, 40, and 45 MPa) were considered. Each design case considered various aspects, namely basic requirements (strength and workability) and ecological aspects (CO_2_ emissions). Multi-objective optimization was implemented based on a composite desirability function that was calculated using the individual responses and desirability for each objective.
(3) From Mix-30 to Mix-45, as the content of supplementary cementing materials decreased, CO_2_ emissions increased. Because the aim of CO_2_ emissions optimization is to reach low CO_2_ emissions, increasing CO_2_ emissions lowered the individual desirability of the cases. In addition, the individual desirability values of strength and flow were 1 for each case. Consequently, the composite desirability decreased from 0.977 to 0.609 as the design strength increased from 30 to 45 MPa. The performance trend of the best combination was consistent with the experimental results.
(4) The results presented in this paper can be used to guide a general method for designing low-carbon concrete. Adopting this method requires two steps. The first step is to produce an experimental design using the response surface method, conduct experimental research on the strength and fluidity, and calculate the CO_2_ emissions. The second step is to optimize the design according to the required strength and flow level and choose the right combination of low-CO_2_ cementitious materials.

## Figures and Tables

**Table 1:** Binders’ chemical compositions.

	Cement (%)	Limestone (%)	Calcined Clay (%)	Slag (%)
SiO_2_	22.1	1.8	63.27	32.2
Al_2_O_3_	5.23	0.19	25.36	15.7
Fe_2_O_3_	3.09	-	7.55	0.65
CaO	62.41	54.14	0.45	38.9
MgO	2.62	1.41	0.66	7.08
Na_2_O	0.09	-	-	0.30
TiO_2_	0.21	-	0.86	0.43
SO_3_	2.32	-	0.12	2.65
LOI ^a^	1.56	41.52	0.94	1.25
Density (g/cm^3^)	3.15	2.60	2.50	2.90

^a^ Loss on ignition.

**Table 2:** Replacement ranges of mineral admixtures.

Component	Name	Minimum	Maximum (%)	Low-Coded	High-Coded
A	Limestone	0.0000	10.00	−1 ↔ 0.00	+1 ↔ 10.00
B	Clay	0.0000	20.00	−1 ↔ 0.00	+1 ↔ 20.00
C	Slag	0.0000	30.00	−1 ↔ 0.00	+1 ↔ 30.00

**Table 3:** BBD of hybrid binders.

Runs	Coded Values				Mass (%)		
	A	B	C	OPC	Limestone (A)	Clay (B)	Slag (C)
M1	−1	−1	−1	100	0	0	0
M2	0	−1	−1	95	5	0	0
M3	−1	0	−1	90	0	10	0
M4	−1	−1	0	85	0	0	15
M5	0	0	−1	85	5	10	0
M6	1	0	−1	80	10	10	0
M7	1	−1	0	75	10	0	15
M8	0	1	−1	75	5	20	0
M9	0	0	0	70	5	10	15
M10	0	0	0	70	5	10	15
M11	0	0	0	70	5	10	15
M12	0	0	0	70	5	10	15
M13	0	0	0	70	5	10	15
M14	1	1	−1	70	10	20	0
M15	0	−1	1	65	5	0	30
M16	−1	1	0	65	0	20	15
M17	−1	0	1	60	0	10	30
M18	1	1	0	55	10	20	15
M19	1	0	1	50	10	10	30
M20	0	1	1	45	5	20	30
M21	1	1	1	40	10	20	30

**Table 4:** CO_2_ emissions of individual components of binders (kg/kg) [27].

Cement	Limestone Powder	Calcined Clay	Slag	Water
0.86	0.008	0.27	0.09	0.0001

**Table 5:** Test results and CO_2_ emissions.

Runs	Strength at 3 Days (MPa)		Strength at 28 Days (MPa)		Flow (mm)		CO_2_ Emissions (kg/m^3^)
	Mean	Standard Deviation	Mean	Standard Deviation	Mean	Standard Deviation	Mean
M1	25.55	0.93	50.40	1.04	247.50	3.39	1052.04
M2	27.21	1.00	49.26	1.05	253.00	4.75	995.84
M3	25.82	1.02	47.83	0.82	210.00	4.89	970.07
M4	22.85	0.88	46.42	0.94	220.50	4.47	906.20
M5	22.87	0.74	48.22	0.92	225.00	4.60	914.76
M6	22.00	0.74	47.29	1.00	228.00	4.57	859.89
M7	19.16	0.78	41.56	1.02	241.00	3.99	795.99
M8	17.74	0.98	45.77	1.04	195.00	4.43	835.28
M9	21.15	0.78	44.99	0.86	213.00	3.62	771.63
M10	21.53	0.97	44.14	1.01	210.10	4.51	771.63
M11	19.84	0.78	44.74	1.00	218.12	3.39	771.63
M12	20.25	1.01	43.04	0.82	217.25	3.80	771.63
M13	20.38	0.81	44.09	0.80	216.30	3.41	771.63
M14	18.96	0.76	46.87	0.94	185.00	3.50	781.27
M15	18.13	0.78	42.52	1.12	236.50	4.71	707.33
M16	16.17	0.90	42.59	0.88	179.00	4.49	747.55
M17	17.31	0.85	43.43	0.98	206.50	3.86	683.52
M18	15.30	0.81	38.34	0.84	170.00	4.92	640.75
M19	16.53	0.98	33.96	1.04	200.00	3.39	576.70
M20	15.32	0.89	33.79	0.85	164.00	4.07	553.97
M21	14.63	0.88	27.41	0.95	160.00	3.97	501.59

**Table 6:** Coefficients of regression of the equations.

Factor	Flow (mm)	3-Day Strength (MPa)	28-Day Strength (MPa)	CO_2_ Emissions (kg/m^3^)
Intercept	213.82	20.55	44.05	771.62
Linear terms				
A—limestone	1.75	−0.8023	−2.62	−54.26
B—clay	−31.50	−2.51	−2.64	−78.48
C—slag	−10.28	−2.98	−4.91	−142.45
Quadratic terms				
A^2^	−5.39	−0.6315	−0.6679	0.2215
B^2^	−4.39	−1.45	−0.9604	0.7804
C^2^	3.47	0.3462	−0.1966	0.7006
Interaction terms				
AB	−5.18	0.6547	0.3993	0.8564
AC	−3.13	0.1745	−2.37	0.8391
BC	−0.6303	1.08	−1.45	1.80
Other terms				
*p*-value	<0.0001	<0.0001	<0.0001	<0.0001
	(significant)	(significant)	(significant)	(significant)
Lack of fit	0.1081	0.1399	0.1836	-
	(not significant)	(not significant)	(not significant)	
R^2^	0.95	0.98	0.975	-

**Table 7:** Objectives of optimization.

Items	Lower Limit	Upper Limit	Goal
Independent variables			
Limestone powder	0	10%	In range
Calcined clay	0	20%	
Slag	0	30%	
Response variables			
Strength at 28 days (MPa)	27.41	50.4	28-day strength ≥ 30, 35, 40, or 45
Flow (mm)	160	253	Flow ≥ 160
CO_2_ emissions (kg/m^3^)	501.59	1052.04	Minimum

**Table 8:** Results of optimal mixtures.

Optimal Combinations	Cement (%)	Limestone (%)	Calcined Clay (%)	Slag (%)
Mix-30	41.19	8.81	20.00	30.00
Mix-35	46.27	3.75	19.98	30.00
Mix-40	53.9	1.03	15.07	30.00
Mix-45	64.26	0.00	5.74	30.00

**Table 9:** Performance of optimal mixtures.

Optimal Combinations	Flow (mm)	28-Day Strength (MPa)	CO_2_ Emissions (kg/m^3^)	Composite Desirability
Mix-30	162.36	30.00	514.09	0.977
Mix-35	171.85	35.00	567.62	0.881
Mix-40	189.36	40.00	633.41	0.761
Mix-45	213.66	45.00	716.63	0.609

**Table 10:** CO_2_ emissions based on 1 kg binder.

	OPC	Limestone (A)	Clay (B)	Slag (C)	CO_2_ Emission for 1 kg Binder (kg/kg)
M1	100	0	0	0	0.86
M2	95	5	0	0	0.8174
M3	90	0	10	0	0.801
M4	85	0	0	15	0.7445
M5	85	5	10	0	0.7584
M6	80	10	10	0	0.7158
M7	75	10	0	15	0.6593
M8	75	5	20	0	0.6994
M9	70	5	10	15	0.6429
M10	70	5	10	15	0.6429
M11	70	5	10	15	0.6429
M12	70	5	10	15	0.6429
M13	70	5	10	15	0.6429
M14	70	10	20	0	0.6568
M15	65	5	0	30	0.5864
M16	65	0	20	15	0.6265
M17	60	0	10	30	0.57
M18	55	10	20	15	0.5413
M19	50	10	10	30	0.4848
M20	45	5	20	30	0.4684
M21	40	10	20	30	0.4258

**Table 11:** Optimization design results (CO_2_ emissions based on 1 kg binder).

Optimal Combinations	Cement (%)	Limestone (%)	Calcined Clay (%)	Slag (%)
Mix-30	41.19	8.81	20.00	30.00
Mix-35	46.40	4.06	19.54	30.00
Mix-40	54.02	1.28	14.70	30.00
Mix-45	64.26	0.00	5.74	30.00

**Table 12:** Performance of optimal mixtures (CO_2_ emissions based on 1 kg binder).

Optimal Combinations	Flow (mm)	28-Day Strength (MPa)	CO_2_ Emissions (kg/kg)	Composite Desirability
Mix-30	162.36	30.00	0.4359	0.977
Mix-35	173.33	35.00	0.479	0.877
Mix-40	190.78	40.00	0.531	0.757
Mix-45	213.66	45.00	0.595	0.610

## Data Availability

The data presented in this study are available in the respective references.
